# Differentiation and Variability in the Rhizosphere and Endosphere Microbiomes of Healthy and Diseased Cotton (*Gossypium* sp.)

**DOI:** 10.3389/fmicb.2021.765269

**Published:** 2021-12-06

**Authors:** Yingwu Shi, Hongmei Yang, Ming Chu, Xinxiang Niu, Ning Wang, Qing Lin, Kai Lou, Changgeng Zuo, Jingyi Wang, Qiang Zou, Yumeng Zhang

**Affiliations:** ^1^Institute of Microbiology, Xinjiang Academy of Agricultural Sciences, Urumqi, China; ^2^Xinjiang Laboratory of Special Environmental Microbiology, Urumqi, China; ^3^Key Laboratory of Agricultural Environment in Northwest Oasis of Ministry of Agriculture and Countryside, Urumqi, China; ^4^Institute of Soil, Fertilizer, and Agricultural Water Conservation, Xinjiang Academy of Agricultural Sciences, Urumqi, China; ^5^College of Life Sciences and Technology, Xinjiang University, Urumqi, China

**Keywords:** endophytic microbiome, rhizosphere microbiome, healthy and diseased, cotton, diversity, Illumina MiSeq

## Abstract

The plant microbiome is a key determinant of health and productivity. However, it is still difficult to understand the structural composition of the bacterial and fungal microbiomes of diseased and healthy plants, especially the spatial dynamics and phylogenies of endophytic and rhizosphere microbial communities. We studied the differentiation and variability in the rhizosphere and endosphere microbiomes of healthy and diseased cotton from north and south of the Tianshan Mountains using the methods of PCR-based high-throughput sequencing and real-time quantitative PCR. The endophytic and rhizosphere bacterial abundances in the diseased plants were greater than those of healthy plants. The numbers of endophytic and rhizosphere fungi associated with diseased plants were greater than those associated healthy plants (*p <* 0.05). Endophytic and rhizosphere bacteria did not share common OTUs. The dominant rhizosphere bacteria were Proteobacteria (29.70%), Acidobacteria (23.14%), Gemmatimonadetes (15.17%), Actinobacteria (8.31%), Chloroflexi (7.99%), and Bacteroidetes (5.15%). The dominant rhizosphere fungi were Ascomycota (83.52%), Mortierellomycota (7.67%), Basidiomycota (2.13%), Chytridiomycota (0.39%), and Olpidiomycota (0.08%). The distribution of dominant bacteria in different cotton rhizosphere soils and roots differed, with the dominant bacteria *Pseudomonas* (15.54%) and *Pantoea* (9.19%), and the dominant fungi *Alternaria* (16.15%) and *Cephalotrichum* (9.10%) being present in the greatest numbers. At sampling points in different ecological regions, the total numbers of cotton endophytic and rhizosphere microbiome OTUs from southern to northern Xinjiang showed an increasing trend. There were significant differences in the composition and diversity of rhizosphere microbes and endophytes during the entire cotton growth period and in representative ecological regions (*p* < 0.01), whereas rhizosphere microbes and endophytes showed no significant differences among the four growth periods and in representative ecological regions. RB41, H16, Nitrospira, and Sphingomonas play important roles in the microbial ecology of cotton rhizosphere soil. Pseudomonas accounted for a large proportion of the microbes in the cotton rhizosphere soil. This study provides an in-depth understanding of the complex microbial composition and diversity associated with cotton north and south of the Tianshan Mountains.

## Introduction

Cotton is an important natural fiber crop worldwide. It grows well at moderate temperatures and high light exposure, and it is suitable for growing in dry areas, such as Xinjiang, China. Endophytic and rhizosphere microbes promote plant growth, improve plant resistance to external adverse environmental conditions as well as diseases and insect pests (Bainard et al., 2016; Beckers et al., 2017; Aghdam and Brown, 2021).

*Verticillium* wilt is a soil-borne disease that infects cotton vascular bundles. The main pathogen is *Verticillium dahliae* Kleb., which can infect cotton (Tjamos et al., 2000; Zhu et al., 2007). It has characteristics of a long survival time in the soil and a complex infection process, making it difficult to control (Tjamos et al., 2000; Zhu et al., 2007).

The community and quantity dynamics of endophyte in cotton have been studied. Misaghi and Donndelinger (1990) isolated six endophytic bacteria from two cotton varieties, and the dominant genus was *Erwinia*. McInroy and Kloepper (1995) isolated 32 genera of endophytic bacteria from cotton rhizomes and analyzed the dynamics of endophytic bacteria in cotton. Lu et al. (1991) found that cotton vascular bacteria are mainly *Bacillus* spp., followed by *Xanthomonas* spp., *Erwinia* spp., and *Chromobacterium* spp. Xia et al. (1996, 1997) used cotton endophytic bacteria to induce cotton’s systemic resistance to *Verticillium* wilt. Li et al. (2009) found that cotton variety, growth period, and organs affect the number of endophytic bacteria in cotton, and endophytic bacteria generally form the dominant population with a high diversity level. Zhou et al. (2012) studied the synergistic control of cotton *Verticillium* wilt by antagonistic endophytes at different cotton growth stages.

The relationships between rhizosphere microorganisms and host plants are very complicated (Egamberdieva et al., 2008). Environmental factors affect the compositions of plant microbial communities (Xu et al., 2014). Rhizosphere microorganisms having biological control potential inhabit the surfaces of plant roots. They also avoid pathogen resistance after the use of chemicals (Varo et al., 2016). The occurrence of plant diseases is closely correlated with the community structure of rhizosphere microbes.

There are many discussions about plant endophytes originating from rhizosphere microorganisms (Hallmann et al., 1997; Cocking, 2003). However, the relationship between endophytes and rhizosphere microorganisms is still unclear. Current studies have shown that the microbiome of cotton changes with space and time and affects availability of soil nitrogen, thereby affecting the growth and reproduction of plant (Zhang et al., 2011; Tian and Zhang, 2017; Moronta-Barrios et al., 2018; Lu et al., 2020; Putrie et al., 2020). Therefore, the changes in microbiome may affect healthy of cotton.

In this study, we evaluate microbiome niche differentiation of microbial communities associated with the rhizosphere soil and the root endosphere of field grown cotton using pyrosequencing in cotton fields from seven typical ecological regions in Xinjiang, China. It was studied to clarify the relationships between rhizosphere microorganisms and endophytes, reveal the spatiotemporal dynamics of rhizosphere microorganisms and endophytes, and clarify the difference between rhizosphere microorganisms and endophytes on diseased and healthy cotton plants.

## Materials and Methods

### Sampling Area Description

This study was mainly carried out on the northern slope and southern foot of the Tianshan Mountains, including seven typical ecological cotton regions, Shihezi, Wusu, Jinghe, Hami, Korla, Alar, and Tumshuk (Supplementary Table 1). Xinjiang is in an arid to semi-arid area, with little rainfall all year round, an arid climate, and abundant light and heat resources.

### Collection of Rhizosphere Soil and Plant Samples

Randomly samples of good growth and uniform size were collected at the cotton seedling, bud, flowering stage, and flocculent stages. Each sample consisted of three randomly selected plants. Samples collected included roots and rhizosphere soil (0–3 mm from the root surface) (Zhang and Kong, 2014). All samples were placed in aseptic bags which placed ice straightway and transported back to our lab. The plant materials were surface-sterilized according to Shi et al. (2021b) method.

### DNA Extraction, and 16S/ITS rRNA Amplification and Sequencing

For the total DNA extraction, and the PCR amplification and sequencing, of the cotton root tissue and rhizosphere soil samples, an EZ-10 Spin Column Plant Genomic DNA Purification Kit (Sangon Biotech, Shanghai, China) and MOBIO Soil DNA Extraction Kit (Qiagen) were used, respectively (Shi et al., 2020, 2021b). DNA samples of sufficient quality were sent to Shanghai Meiji Biomedical Technology Co., Ltd. for PCR amplification and high-throughput sequencing. The bacterial 16S rRNA universal primer pairs 799F/1115 and 338f/806r were selected to amplify the 16S rRNA sequences of endophytic and rhizospheric bacteria (Shi et al., 2020, 2021b). The fungal ITS rRNA universal primer pairs ITS1F/ITS2 and ITS1F/ITS4 were selected to amplify the ITS rRNA sequences of endophytic and rhizospheric fungi (Shi et al., 2021a). High-throughput sequencing was performed using Illumina’s MiSeq sequencing platform.

### Data Processing and Bioinformatics Analysis

After the sequencing was completed, the Fast Length Adjustment of Short reads software for sequence splicing was used in accordance with the overlap between PE reads, and then effective sequences were obtained after Tag filtering and Tag de-chimerism. The UPARSE software was used to perform OTU clustering and species diversity analyses on the obtained effective sequences on the basis of a 97% similarity level (Tian and Zhang, 2017; Lu et al., 2020; Zhu et al., 2020). Mothur and Qiime software packages were used to calculate the biodiversity index of the microbiome of cotton based on the OTU classification level. This included the ACE, Chao, Shannon, and Simpson indices (Lopez-Garcia et al., 2018). Through hierarchical cluster and principal component analyses of cotton organ samples, the similarities and differences in microbiome community compositions among different organ samples were explored.

## Results

### Variability in the Community Diversity of Cotton Rhizosphere and Endophytic Bacteria and Fungi

**FIGURE 1:**
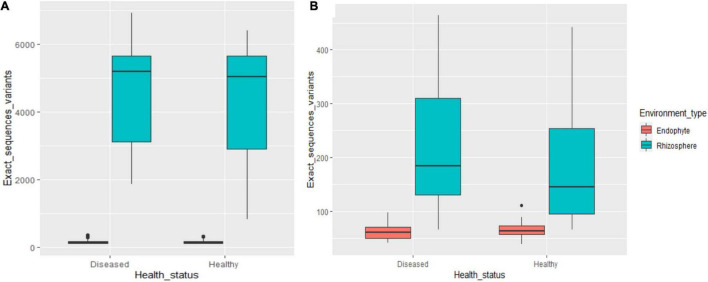
The number of OTUs of rhizosphere and endophytic bacteria **(A)** and fungi **(B)** in healthy and diseased cotton (*P* < 0.05).

The microbiome abundance levels in the rhizosphere soil and root tissues of diseased plants are greater than those of healthy plants, but the fungal abundance in the root tissues of diseased plants was less than that of healthy plants (Figures 1A,B). At the OTU and species levels, there are significant differences between endophytic fungi and rhizosphere fungi in the diseased and healthy cotton plants, while the differences in bacteria are not significant.

**FIGURE 2:**
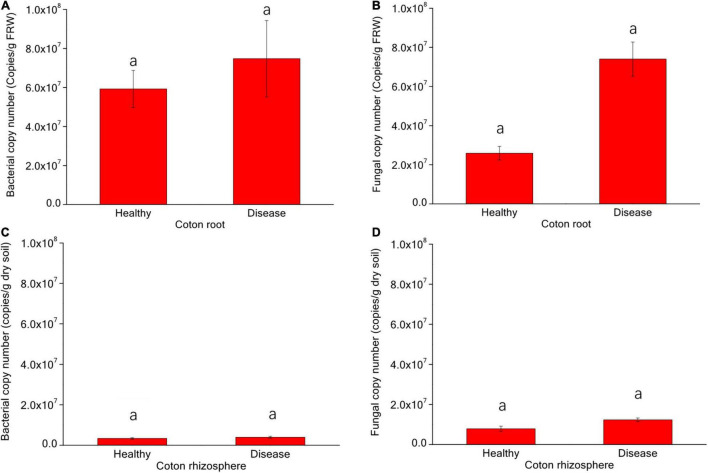
Variation of numbers (**A** endophytic bacteria; **B** endophytic fungi; **C** rhizosphere bacteria; and **D** rhizosphere fungi) of rhizosphere and endophytic bacteria and fungi in healthy and diseased cotton (*P* > 0.05).

The q-PCR results showed that there were significant differences in the numbers of endophytic and rhizosphere microbiomes from healthy and diseased cotton (Figures 2A–D). The number of endophytic bacteria in diseased plants was higher than in healthy plants, while the rhizosphere bacteria of healthy cotton plants are higher than diseased plants (*p* > 0.05). The number of endophytic and rhizosphere fungi from diseased plants was higher than from healthy plants (*p* > 0.05).

**FIGURE 3:**
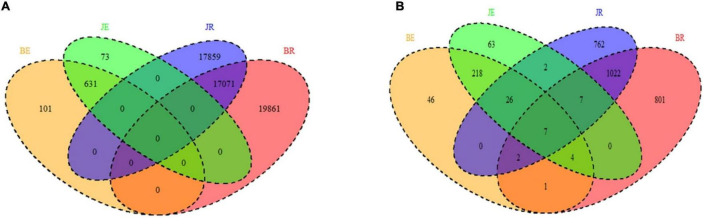
Venn diagram showing the unique and shared OTUs (3% distance level) (**A** bacteria; **B** fungi) in the rhizosphere soil and root of cotton by different niche and pathological statuses (endophytic diseased, endophytic healthy, rhizosphere diseased, and rhizosphere healthy, respectively). BE, JE, BR, and JR represent endophytic diseased, endophytic healthy, rhizosphere diseased, and rhizosphere healthy, respectively.

As shown in Figures 3A,B, endophytic and rhizospheric bacteria from diseased and healthy cotton do not represent the same OTUs, whereas endophytic and rhizospheric fungi are from seven identical OTUs. As shown in Table 1, at the OTU level, there are significant differences in the numbers of the rhizosphere and endophytic microbe OTUs from diseased and healthy cotton (*p* < 0.05; Tables 2, 3).

**TABLE 1:** Analysis of similarity (ANOSIM) of the difference at Phylum and OUT among samples associated with *Gossypium* sp.

Phylogenetic level	Phylum		OTU	
	*R*	*P*	*R*	*P*
Bacteria	0.35	0.001**	0.192601	0.001**
Fungi	0.24	0.001**	0.14221	0.001**

Plant compartment effects on the bacterial community structures were calculated using ANOSIM (analysis of similarities) with the Spearman rank correlation method in Primer 7 (10,000 permutations). Plant compartments (rhizosphere soil, root, stem, and leaf) were a priori defined groups at two phylogenetic levels: phylum level and OTU level. Significance levels: *P ≤ 0.01; **P ≤ 0.001; ***P ≤ 0.0001. R, ANOSIM test statistic. Graphical results of ANOSIM are displayed in Additional file.

**TABLE 2:** Adonis analysis of the difference among bacteria in samples associated with *Gossypium* sp.

	Df	SumsOfSqs	MeanSqs	F.Model	R2	Pr(>F)
group_factor$BJRE	3	9.110279	3.03676	8.587604	0.192601	0.001
Residuals	108	38.1911	0.353621	–	0.807399	–
Total	111	47.30138	–	–	1	–

*The larger the value of R2 (the ratio of group variance to total variance), the more significant the differences were among the organs and habitats. P < 0.05 indicates a high reliability of the test.*

**TABLE 3:** Adonis analysis of the difference among samples fungi in associated with *Gossypium* sp.

	Df	SumsOfSqs	MeanSqs	F.Model	R2	Pr(>F)
group_factor$BJRE	3	7.064223	2.354741	5.968319	0.14221	0.001
Residuals	108	42.61033	0.39454	–	0.85779	–
Total	111	49.67456	–	–	1	–

### Differences in the Composition of Cotton Rhizosphere and Endophytic Microbiome

**FIGURE 4:**
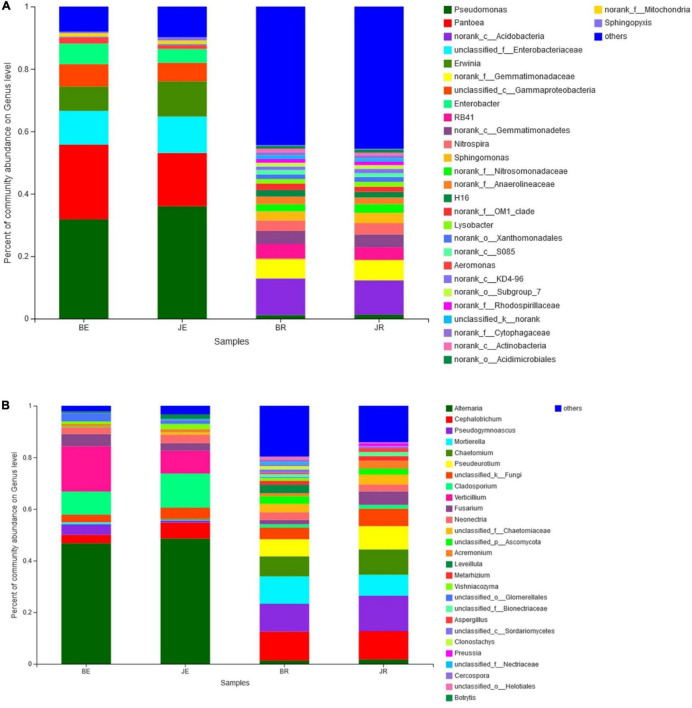
Bacteria **(A)** and Fungal **(B)** composition of the different communities at the genera level. Relative read abundance of different bacterial phyla within the different communities. Phyla, classes, and genera making up less than 1% of total composition in all three libraries were classified as “others”.

At the phylum level, there were 14 bacterial phyla present in the two communities, 4 endobacteria and 14 rhizobacteria. Proteobacteria represent the most abundant phyla (60.6% of the total readings), followed by Acidobacteria (11.6%), Gemmatimonadetes (7.6%), Actinobacteria (6.9%), Chloroflexi (4.0%), Bacteroidetes (2.9%), Nitrospirae (1.8%), Firmicutes (1.4%), and Planctomycetes (1.0%). Proteobacteria are composed of alpha-, beta-, and gamma-proteobacteria (Figure 4A). There were no common OTUs between the endobacteria and rhizobacteria. The dominant rhizosphere flora were Proteobacteria (29.70%), Acidobacteria (23.14%), Gemmatimonadetes (15.17%), Actinobacteria (8.31%), Chloroflexi (7.99%), Bacteroidetes (5.15%), Nitrospirae (3.67%), Planctomycetes (2.07%), and Verrucomicrobia (1.16%). Among root endophytic bacteria, Proteobacteria (91.41%), Actinobacteria (5.49%), Firmicutes (2.10%), and Bacteroidetes (0.67%) represented the most commonly observed OTUs (Figure 4A).

There were six fungal phyla in two communities, six phyla of rhizosphere fungi, and four phyla of endophytic fungi. Ascomycota was the most abundant (88.66% of the total reading), followed by Mortierellomycota (4.28%), Basidiomycota (2.26%). Ascomycota is composed of Sordariomycetes, Dothideomycetes, and Leotiomycetes (Figure 4B). The dominant rhizosphere flora were Ascomycota (83.52%), Mortierellomycota (7.67%), Basidiomycota (2.13%). Among root endophytic fungi, Ascomycota (93.80%), Basidiomycota (2.38%), Mortierellomycota (0.90%), and represent the most commonly observed OTUs (Figure 4B).

Consistent with these observations, the nonparametric analyses of variance of the 16S and ITS data confirmed the dissimilarities of the rhizosphere and endophytic microbial communities (Pr = 0.001, *R*^2^ = 1.0000; Pr = 0.001, *R*^2^ = 0.9798).

In general, the distributions of dominant bacteria in different cotton rhizosphere soils and roots (average relative abundance > 0.1%) were different, with the bacteria *Pseudomonas* (15.54%) and *Pantoea* (9.19%), and fungi *Alternaria* (16.15%) and *Cephalotrichum* (9.10%) existing in the largest numbers (Figures 4A,B).

### Spatiotemporal Dynamics of Bacterial and Fungal Communities in Cotton Rhizospheres and Roots

**FIGURE 5:**
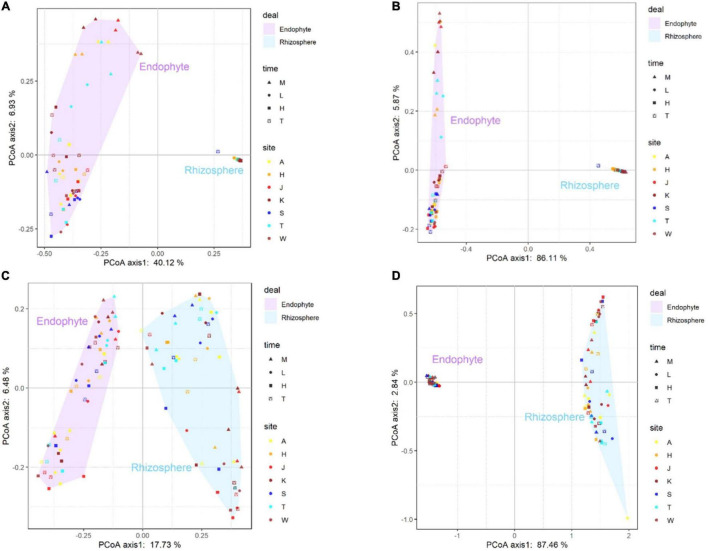
The principal-coordinate analysis (PCoA) plots were based on Bray-Curtis **(A)** and weighted-unfrac **(B)** dissimilarity of bacterial **(A,B)** and fungal **(C,D)** communities in sampling site (A, K, T, H, S, W, and J represent the Alaer, Kuerle, Tumushuke, Hami, Shihezi, Wusu, and Jinghe, respectively) and growing season (M, L, H, and T represent seedling, bud, flowering, and boll opening stages, respectively) Differentiation in the rhizosphere and endosphere microbiomes was significantly (PERMANOVA *p <* 0.05).

The developmental stage explained 91.98% of the rhizosphere and endophytic bacteria using the weighted UniFrac distance, but explained only 47.05% of the rhizosphere and endophytic bacteria using the Bray-Curtis distance (Figures 5A,B). In addition, the developmental stage explained 90.30% of the rhizosphere and endophytic fungi using the weighted UniFrac distance, but explained only 24.21% of the rhizosphere and endophytic fungi using the Bray-Curtis distance (Figures 5C,D). As the developmental stage changed, the total number of endophytic bacterial OTUs in cotton first increased and then decreased, and the total number of OTUs in the rhizosphere decreased. The total number of endophytic fungal OTUs decreased and then increased, and the total number of rhizospheric fungal OTUs increased (Supplementary Figures 1A,B). With a change in the developmental stage, the total number of endophytic microbiomes OTUs in diseased and healthy cotton plants decreased, while the total number of OTUs in the rhizosphere of diseased and healthy cotton plants first decreased and then increased (Supplementary Figures 2A,B).

During the entire cotton growth period and in representative ecological regions, there were significant differences in the numbers of rhizospheric and endophytic pathogens as assessed by qPCR (*p* < 0.01; Supplementary Figures 3A,B). There were significant differences in the community compositions of cotton rhizosphere bacteria and endophytic fungi at different developmental stages (Supplementary Figures 3A,B). An analysis revealed that the relative abundances of microorganisms on diseased and healthy plants were quite different, and they were mainly distributed in the three bacterial phyla, Proteobacteria (60.27 and 58.81%, respectively), Acidobacteria (11.89 and 11.80%, respectively), and Gemmatimonadetes (6.25 and 7.03%, respectively), and two fungal genera, Verticillium (5.43 and 2.98%, respectively) and Acremonium (1.03 and 2.44%, respectively) (Supplementary Figures 4A,B).

At sampling points in different ecological regions, the total numbers of rhizosphere and endophytic bacteria OTUs in cotton from southern to northern Xinjiang showed an increasing trend (Supplementary Figures 5A,B). The total number of rhizosphere and endophytic fungi OTU is basically the same at sampling points. At sampling points in different ecological regions, only the number of endophytic fungi in healthy plants is reduced, the rest of the number of rhizosphere and endophytic microbiomes increasing in diseased and healthy cotton plants from southern to northern Xinjiang (Supplementary Figures 5A,B).

### Networks and Phylogeny in the Rhizosphere and Endophytic Microbiomes of Diseased and Healthy Cotton Plants

**FIGURE 6:**
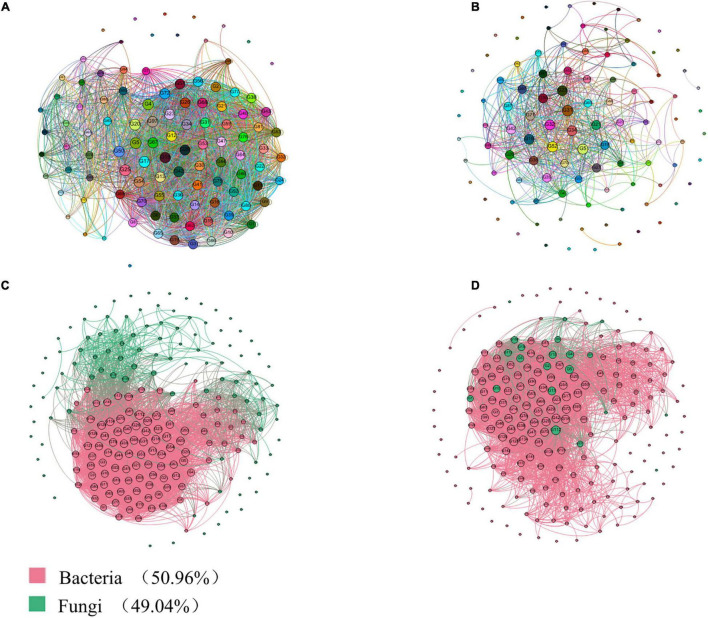
Correlation network analysis of the rhizosphere and endosphere microbiomes in *Gossypium* sp. based on genus level. **(A)** Interaction analysis of bacteria; **(B)** Interaction analysis of fungi. **(C)** Interaction analysis of bacteria and fungi; **(D)** Interaction analysis of core microbiota-common microbiota. The size of each node is proportional to the degree.

As shown in Figure 6A, 44 nodes representing genera RB41, H16, Nitrospira, Sphingomonas Steroidobacter, Aeromonas, Lysobacter, and Erwinia are closely related to other bacterial genera, and their degrees of connection are the highest among all the nodes. Lysobacter, which are dominant in relative abundance in the rhizosphere and internal environment, occupy important positions in the microbial ecology of cotton rhizosphere soil and have very important impacts on other bacterial groups. In addition, as shown in Figure 6A, Erwinia accounts for a large proportion of the microbes in the cotton rhizosphere soil, which is consistent with the bacterial community composition (Figure 6A). In Figure 6A, RB41 and norank_c__Acidobacteria belong to Acidobacteria, H16, unclassified_f__Enterobacteriaceae and Sphingomonas belong to Proteobacteria, and Nitrospira belongs to Nitrospirae, with a higher total relative abundance. RB41, H16, Nitrospira, and Sphingomonas also play important roles in the composition of the rhizosphere soil bacterial community in cotton fields.

As shown in Figure 6B, the two nodes representing the genera *Alternaria*, *Cephalotrichum*, *Pseudogymnoascus*, *Mortierella*, *Chaetomium*, *Pseudeurotium*, *unclassified_k__Fungi*, *Cladosporium*, and *Verticillium* are closely related to other true genera, and their degrees of connection are the highest at all the nodes. RB41, H16, Nitrospira, norank_c__Acidobacteria, unclassified_f__Enterobacteriaceae, norank_f__OM1_clade, and Sphingomonas, which are dominant in relative abundance in the rhizosphere and internal environments and have nitrogen transformation abilities, occupy important positions in the microbial ecology of cotton rhizosphere soil and have a very important impacts on other bacterial groups.

An indicator species analysis was used to identify the sensitive OTUs associated with each healthy group, and their positions in the soil microbial community were shown by constructing a co-occurrence network (Figures 6C,D). Three sensitive OTUs correlated with cotton plant health were defined as the cornerstones of the network. The most detailed classifications were the bacteria *Pseudomonas*, *Bacillus*, and *Serratia*, as well as the fungi *Alternaria*, *Cladosporium*, *Cephalotrichum*, *Acremonium*, *Chaetomium*, *Stemphylium*, *Premium*, and *Stachybotrys*. In addition, we visualized two modules showing a high interactivity in the network, with the largest module being regarded as the central module.

**FIGURE 7:**
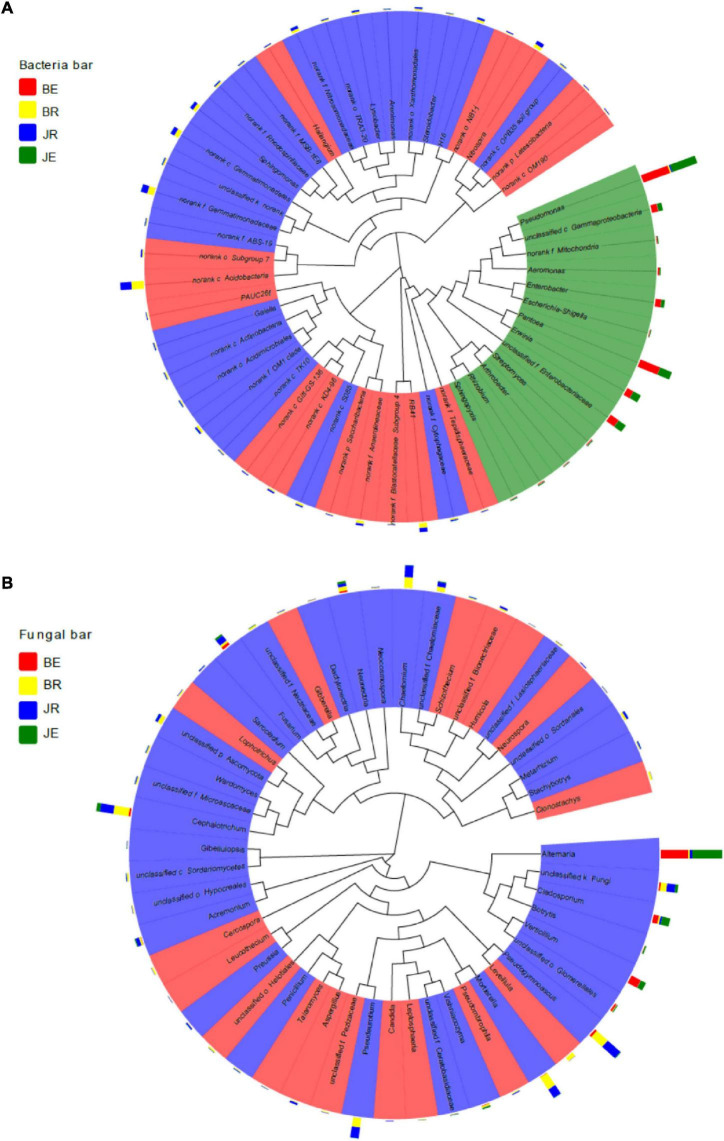
A Phylogenetic tree of the top 50 bacterial **(A)** and fungal **(B)** genera and their distributions in the rhizosphere soils and root of cotton with different pathological statuses. The background color of the taxonomic designation represent its classification at the genus level. The bar plot exhibits the relative abundance of the genus in the BE, JE, BR, and JR samples. BE, JE, BR, and JR, respectively, represent endophytic diseased, endophytic healthy, rhizosphere diseased, rhizosphere healthy, respectively.

As shown in Figure 7, rhizosphere and endophytic bacteria are phylogenetically significantly different, whereas rhizospheric and endophytic fungi are phylogenetically similar. Among the bacteria, *Proteobacteria* and *Actinobacteria* are the dominant genera, among endophytic fungi, *Alternaria* is the dominant genus, and among rhizospheric fungi, *Pseudogymnoascus*, *Mortierella*, *Pseudeurotium*, and *Cephalotrichum* are the dominant genera.

## Discussion

### The Microbiome Differentiation Between the Cotton Rhizosphere and the Endogenous Environment

In this study, cotton rhizosphere and endophytic microbiomes were significantly different at the OTU level, unsimilar to previous reports (Beckers et al., 2017; Qiao et al., 2017; Tian and Zhang, 2017; Huang, 2018). Endophytes are almost ubiquitous in different plants. Their original sources are thought to be the epiphytic flora of plant rhizospheres or root surfaces, leaf surfaces, internal seed tissues, and tissue-cultured seedlings. Some studies believe that rhizosphere soil is the primary colonization source of endophytes. Endophytes exist in the roots, stems, branches, and leaves of plants, and they also colonize the xylem and between cells. There are limited reports of endophytes colonizing cells (Dong et al., 2003).

Cheng (2018) found that the tomato microbiome is mainly composed of three groups: Proteobacteria, Actinobacteria, and Firmicutes. Among them, Proteobacteria is the main dominant member of the tomato root microbiome, accounting for 34.15 and 39.18% of the total number of tomato rhizosphere and root endophytic bacteria, respectively. The structures and compositions of the endophytic and rhizosphere bacteria of tomato root are different, and this is mainly manifested by the high enrichment of *Pseudomonas* and *Bacillus* in rhizosphere endophytes. Some common plant probiotics, such as *Bacillus* and *Pseudomonas*, account for small proportions of rhizosphere bacteria, whereas *Pseudomonas* is the main dominant group of endophytes. This was similar to our findings.

The microbial communities in the two microenvironments, cotton root tissue and rhizosphere soil, are significantly different, indicating that the functions of the two microenvironments differ. Most of the endophytic fungi in healthy and diseased cotton plants belong to imperfect fungi and Ascomycota. The number of *Verticillium* fungi increased in the middle and late stages of cotton growth, indicating that *V. dahliae* infection affects these stages.

### Soil Is the Decisive Factor for the Cotton Rhizosphere Microbial Community

Various factors, such as soil pH, water content, electrical conductivity, and nutrient contents, including alkali-hydrolyzable nitrogen and available phosphorus, are related to changes in soil bacterial community structures (Preem et al., 2012; Yang et al., 2018). Liu et al. (2021) showed that the physical and chemical properties of soil have significant impacts on bacterial community diversity levels and population structures, whereas field diseases have no significant impacts on soil bacterial community diversity levels and population structures.

Here, the physical and chemical properties of the soil were responsible for the significant differences in the bacterial community structures in the rhizosphere soil between healthy and diseased cotton plants. Soil pH and total salt content were the main factors affecting the bacterial community diversity and population structure. The pH value was significantly positively correlated with bacterial community diversity, whereas total salt was significantly negatively correlated with bacterial community diversity. These conclusions are consistent with those of previous studies, and total nitrogen, total phosphorus, and organic matter were not significantly correlated with bacterial community diversity. Marschner et al. (2001) also found that nitrogen fertilizer has no significant effects on soil bacterial community structures. Thus, the conclusions of this study are consistent with those of previous studies.

Based on previous studies, soil physical and chemical properties, soil types, and soil moisture are the main factors affecting soil bacterial communities (Kuramae et al., 2012; Qiao et al., 2017). There are some controversies about the influence of plant species, crop rotation, and other factors on the structures of soil microbial communities. The reduction in soil-borne diseases is correlated with the decrease in pathogenic bacteria in the soil, the decrease in the pathogenicity of pathogenic bacteria or the increase in the beneficial microorganism’s population (Bailey and Lazarovits, 2003; Bonanomi et al., 2010).

At the genus level, the abundances of the beneficial bacteria *Lysobacter* and *Pseudomonas* in the soil of severely diseased fields in Aksu were 3.67 and 3.15%, respectively, which were significantly greater than those in the non-diseased fields (2.35 and 0.18%, respectively). However, the abundances of *Bacillus* and *Streptomyces* were not significantly different between the severely diseased fields in Aksu, Shihezi, and Korla and the control fields (Liu et al., 2021). Differential marker analyses also found that *Pseudomonadales* were significantly enriched in the seriously diseased fields in Aksu, whereas *Actinobacteria* were significantly enriched in the severely diseased fields in Shihezi. A correlation analysis showed that the number of OTUs and the alpha-diversity index of cotton soil bacteria are positively correlated with soil pH and total potassium content, and negatively correlated with total salt content, carbon–nitrogen ratio, and organic matter, total nitrogen, and total phosphorus contents, as well as cotton field diseases (Liu et al., 2021). There is no obvious correlation between the degrees, and the soil pH value and total salt content are the main factors affecting the diversity of soil bacteria (Liu et al., 2021).

### Microbial Communities in the Cotton Rhizosphere and Endogenous Environment During Different Growth Periods

Most of the endophytic bacteria in healthy and diseased cotton plants belonged to the phylum Proteobacteria, and the diversity of the community structure was closely correlated with the growth and development of cotton (Zhang et al., 2011). Healthy cotton plants were more abundant than diseased cotton plants, indicating that the invasion of *V. dahliae* affected the endophytes. The number of endophytic bacteria in healthy cotton plants during the same period was greater than in diseased cotton plants (Zhang et al., 2011).

The results of the study showed that most of the endophytic fungi in healthy cotton plants and diseased cotton plants belonged to imperfect fungi and Ascomycota. In the middle and late stages of cotton growth, the number of Verticillium fungi increased, indicating that the infection of *Verticillium dahliae* can affect the diversity of endophytic fungal community structure in the middle and late stages of diseased cotton plants. Research by Liu et al. (2016) showed that *V. dahliae* infections reduce the diversity of endophytic bacteria in cotton plants and increase the diversity of the endophytic fungal community.

In addition, the abundance of rhizosphere microorganisms also plays an important role in maintaining plant health. This study found that the 16S rRNA gene copy number associated with the healthy plants was significantly higher than that of the diseased plants, and the 16S rRNA gene copy number of the rhizosphere bacteria was positively correlated with the health of the plants, which is consistent with previous studies.

The copy number of the 16S rRNA gene of rhizosphere bacteria on healthy cotton is significantly higher than that on the diseased plants. *Verticillium* wilt is always accompanied by a substantial increase in the copy number of the rhizosphere fungal ITS region. The severity of the disease is correlated with the fungal ITS region, and the ratio of ITS copy number to bacterial 16S rRNA gene copy number is significantly positively correlated with disease occurrence (*p* < 0.001). The bacterial community in the rhizosphere soil has an inhibitory effect on the occurrence of cotton *Verticillium* wilt, whereas the fungal community of the rhizosphere soil was conducive to the occurrence of *Verticillium* wilt.

This study overcame the limitations of traditional culture methods and provides a fast, reliable and reproducible method for analyzing changes in rhizosphere microbial abundance. Using this technology, through the absolute quantification of the rhizosphere bacteria and fungi between adjacent plants and the detection of the pathogenic microsclerotia, it is clear that the severity of *Verticillium* wilt in cotton increases significantly with the increase in the number of microsclerotia in the soil (*p* < 0.05).

### Distributions of Microorganisms in Plant Microenvironments

The endophyte microbiome is obviously different from the rhizosphere microbiome, which is the result of the plant niche effect. However, endophytes are closely related to rhizosphere microorganisms.

The cumulative relative abundance of OTUs belonging to modules in soil was significantly (*p* < 0.01) higher than that in roots. In addition, different modules contained different bacterial and fungal phyla (Figure 2C), indicating that different ecological groups have specific microbial lineages.

### Drivers of Microbiome Niche Differentiation

The driving factors behind the differences in microbial niches are the differences in root exudates and internal plant environments (Beckers et al., 2017). A 16S amplicon analysis of the bacterial community structures in different niches, including rhizosphere soil, roots, stems, and leaves, was performed. Compared with that of endophytes, the structure of the rhizosphere microbiome is more stable. Not only do the differences between the rhizosphere and roots need to be further analyzed, but also the endophytes in the stems and leaves. Each plant also has an independent ecological niche, and the core bacteria in different niches of poplars have been identified (Beckers et al., 2017). Understanding the complex host–microbe interactions of cotton provides a theoretical basis for explaining prokaryotic- and eukaryotic-related phytoremediation applications, sustainable agriculture, and secondary metabolite production.

## Conclusion

The dominant rhizospheric microflora were Proteobacteria, Acidobacteria, Gemmatimonadetes, Actinobacteria, Chloroflexi, Bacteroidetes, and Ascomycota, Mortierellomycota, Basidiomycota. The abundance of endophytic and rhizosphere bacteria and fungi in diseased cotton plants is higher than that in healthy plants. Endophytic and rhizospheric bacteria did not share common OTUs. The distribution of dominant bacteria in different cotton rhizosphere soils and roots differed, with the dominant bacteria Pseudomonas and Pantoea, and the dominant fungi Alternaria and Cephalotrichum being present in the greatest numbers. At sampling points in different ecological regions, the total numbers of cotton endophytic and rhizosphere microbiome OTUs from southern to northern Xinjiang showed an increasing trend. There were significant differences in the composition and diversity of rhizosphere microbes and endophytes during the entire cotton growth period and in representative ecological regions, whereas rhizosphere microbes and endophytes showed no significant differences among the four growth periods and in representative ecological regions. Pseudomonas accounted for a large proportion of the microbes in the cotton rhizosphere soil. RB41, H16, Nitrospira, Sphingomonas also play important roles in the rhizosphere soil.

## Data Availability Statement

The original contributions presented in the study are publicly available. This data can be found here: https://www.ncbi.nlm.nih.gov/bioproject/, PRJNA762464.

## Author Contributions

YS designed the work and wrote the manuscript. HY and MC collected the data. XN, NW, QL, KL, CZ, JW, QZ, and YZ edited the manuscript. All authors approved the final version of this manuscript.

## Conflict of Interest

The authors declare that the research was conducted in the absence of any commercial or financial relationships that could be construed as a potential conflict of interest.

## Publisher’s Note

All claims expressed in this article are solely those of the authors and do not necessarily represent those of their affiliated organizations, or those of the publisher, the editors and the reviewers. Any product that may be evaluated in this article, or claim that may be made by its manufacturer, is not guaranteed or endorsed by the publisher.
