# Supplementary material for: Differentiation and Variability in the Rhizosphere and Endosphere Microbiomes of Healthy and Diseased Cotton (Gossypium sp.)
Source: Front Microbiol. 2021 Dec 6;12:765269. doi: 10.3389/fmicb.2021.765269 (PMC8685383; doi:10.3389/fmicb.2021.765269)
Supplement: Supplementary file 1 [file Table_1.docx]

**Table S1** Cotton Variety, locations, elevations, pH and soil types

| Samples | Number of sample | Variety | Soil  classification | Soil pH | Latitude | longitude | Elevation (m) | The growth period | Locales |
| --- | --- | --- | --- | --- | --- | --- | --- | --- | --- |
| AMJT | 3 | Zhong 35 | irrigated desert soil | 8.20 | 40°20'19.90"N | 81°11'2.76" E | 998 | seedling stage | Alaer |
| ALJT | 3 | Zhong 35 | irrigated desert soil | 8.17 | 40°20'19.90"N | 81°11'2.76" E | 998 | bud stage | Alaer |
| AHJT | 3 | Zhong 35 | irrigated desert soil | 8.12 | 40°20'19.90"N | 81°11'2.76" E | 998 | flowering stage | Alaer |
| ATJT | 3 | Zhong 35 | irrigated desert soil | 8.08 | 40°20'19.90"N | 81°11'2.76" E | 998 | boll-opening stage | Alaer |
| KMJT | 3 | Zhong 49 | desert grey soil | 7.97 | 40°27'55.80" N | 87°7'53.40"E | 851 | seedling stage | Kuerle |
| KLJT | 3 | Zhong 49 | desert grey soil | 8.14 | 40°27'55.80" N | 87°7'53.40"E | 851 | bud stage | Kuerle |
| KHJT | 3 | Zhong 49 | desert grey soil | 8.18 | 40°27'55.80" N | 87°7'53.40"E | 851 | flowering stage | Kuerle |
| KTJT | 3 | Zhong 49 | desert grey soil | 8.12 | 40°27'55.80" N | 87°7'53.40"E | 851 | boll-opening stage | Kuerle |
| TMJT | 3 | Xinluzhong 60 | alluvial soil | 8.03 | 39°55'12.50" N | 79°7'41.81" E | 1091 | seedling stage | Tumushuke |
| TLJT | 3 | Xinluzhong 60 | alluvial soil | 8.13 | 39°55'12.50" N | 79°7'41.81" E | 1091 | bud stage | Tumushuke |
| THJT | 3 | Xinluzhong 60 | alluvial soil | 8.13 | 39°55'12.50" N | 79°7'41.81" E | 1091 | flowering stage | Tumushuke |
| TTJT | 3 | Xinluzhong 60 | alluvial soil | 8.07 | 39°55'12.50" N | 79°7'41.81" E | 1091 | boll-opening stage | Tumushuke |
| HMJT | 3 | Mihe 1 | brown desert soil | 7.87 | 42°54'52.09" N | 93°16'14.99" E | 875 | seedling stage | Hami |
| HLJT | 3 | Mihe 1 | brown desert soil | 8.08 | 42°54'52.09" N | 93°16'14.99" E | 875 | bud stage | Hami |
| HHJT | 3 | Mihe 1 | brown desert soil | 8.25 | 42°54'52.09" N | 93°16'14.99" E | 875 | flowering stage | Hami |
| HTJT | 3 | Mihe 1 | brown desert soil | 8.4 | 42°54'52.09" N | 93°16'14.99" E | 875 | boll-opening stage | Hami |
| SMJT | 3 | 15-19 | desert grey soil | 8.20 | 44°20'6.29"N | 86°2'51.76" E | 421 | seedling stage | Shihezi |
| SLJT | 3 | 15-19 | desert grey soil | 8.14 | 44°20'6.29"N | 86°2'51.76" E | 421 | bud stage | Shihezi |
| SHJT | 3 | 15-19 | desert grey soil | 8.17 | 44°20'6.29"N | 86°2'51.76" E | 421 | flowering stage | Shihezi |
| STJT | 3 | 15-19 | desert grey soil | 7.92 | 44°20'6.29"N | 86°2'51.76" E | 421 | boll-opening stage | Shihezi |
| WMJT | 3 | Luyan 34 | irrigation cultivation soil | 8.03 | 44°0'44.00" N | 87°21'40.20" E | 375 | seedling stage | Wusu |
| WLJT | 3 | Luyan 34 | irrigation cultivation soil | 8.03 | 44°0'44.00" N | 87°21'40.20" E | 375 | bud stage | Wusu |
| WHJT | 3 | Luyan 34 | irrigation cultivation soil | 8.25 | 44°0'44.00" N | 87°21'40.20" E | 375 | flowering stage | Wusu |
| WTJT | 3 | Luyan 34 | irrigation cultivation soil | 8.21 | 44°0'44.00" N | 87°21'40.20" E | 375 | boll-opening stage | Wusu |
| JMJT | 3 | Hexin 26 | desert grey soil | 8.15 | 44°34'57.07"N | 82°24'37.26" E | 326 | seedling stage | Jinghe |
| JLJT | 3 | Hexin 26 | desert grey soil | 8.24 | 44°34'57.07"N | 82°24'37.26" E | 326 | bud stage | Jinghe |
| JHJT | 3 | Hexin 26 | desert grey soil | 8.3 | 44°34'57.07"N | 82°24'37.26" E | 326 | flowering stage | Jinghe |
| JTJT | 3 | Hexin 26 | desert grey soil | 8.08 | 44°34'57.07"N | 82°24'37.26" E | 326 | boll-opening stage | Jinghe |
| AMBT | 3 | Zhong 35 | irrigated desert soil | 8.20 | 40°20'19.90"N | 81°11'2.76" E | 998 | seedling stage | Alaer |
| ALBT | 3 | Zhong 35 | irrigated desert soil | 8.17 | 40°20'19.90"N | 81°11'2.76" E | 998 | bud stage | Alaer |
| AHBT | 3 | Zhong 35 | irrigated desert soil | 8.12 | 40°20'19.90"N | 81°11'2.76" E | 998 | flowering stage | Alaer |
| ATBT | 3 | Zhong 35 | irrigated desert soil | 8.08 | 40°20'19.90"N | 81°11'2.76" E | 998 | boll-opening stage | Alaer |
| KMBT | 3 | Zhong 49 | desert grey soil | 7.97 | 40°27'55.80" N | 87°7'53.40"E | 851 | seedling stage | Kuerle |
| KLBT | 3 | Zhong 49 | desert grey soil | 8.14 | 40°27'55.80" N | 87°7'53.40"E | 851 | bud stage | Kuerle |
| KHBT | 3 | Zhong 49 | desert grey soil | 8.18 | 40°27'55.80" N | 87°7'53.40"E | 851 | flowering stage | Kuerle |
| KTBT | 3 | Zhong 49 | desert grey soil | 8.12 | 40°27'55.80" N | 87°7'53.40"E | 851 | boll-opening stage | Kuerle |
| TMBT | 3 | Xinluzhong 60 | alluvial soil | 8.03 | 39°55'12.50" N | 79°7'41.81" E | 1091 | seedling stage | Tumushuke |
| TLBT | 3 | Xinluzhong 60 | alluvial soil | 8.13 | 39°55'12.50" N | 79°7'41.81" E | 1091 | bud stage | Tumushuke |
| THBT | 3 | Xinluzhong 60 | alluvial soil | 8.13 | 39°55'12.50" N | 79°7'41.81" E | 1091 | flowering stage | Tumushuke |
| TTBT | 3 | Xinluzhong 60 | alluvial soil | 8.07 | 39°55'12.50" N | 79°7'41.81" E | 1091 | boll-opening stage | Tumushuke |
| HMBT | 3 | Mihe 1 | brown desert soil | 7.87 | 42°54'52.09" N | 93°16'14.99" E | 875 | seedling stage | Hami |
| HLBT | 3 | Mihe 1 | brown desert soil | 8.08 | 42°54'52.09" N | 93°16'14.99" E | 875 | bud stage | Hami |
| HHBT | 3 | Mihe 1 | brown desert soil | 8.25 | 42°54'52.09" N | 93°16'14.99" E | 875 | flowering stage | Hami |
| HTBT | 3 | Mihe 1 | brown desert soil | 8.4 | 42°54'52.09" N | 93°16'14.99" E | 875 | boll-opening stage | Hami |
| SMBT | 3 | 15-19 | desert grey soil | 8.20 | 44°20'6.29"N | 86°2'51.76" E | 421 | seedling stage | Shihezi |
| SLBT | 3 | 15-19 | desert grey soil | 8.14 | 44°20'6.29"N | 86°2'51.76" E | 421 | bud stage | Shihezi |
| SHBT | 3 | 15-19 | desert grey soil | 8.17 | 44°20'6.29"N | 86°2'51.76" E | 421 | flowering stage | Shihezi |
| STBT | 3 | 15-19 | desert grey soil | 7.92 | 44°20'6.29"N | 86°2'51.76" E | 421 | boll-opening stage | Shihezi |
| WMBT | 3 | Luyan 34 | irrigation cultivation soil | 8.03 | 44°0'44.00" N | 87°21'40.20" E | 375 | seedling stage | Wusu |
| WLBT | 3 | Luyan 34 | irrigation cultivation soil | 8.03 | 44°0'44.00" N | 87°21'40.20" E | 375 | bud stage | Wusu |
| WHBT | 3 | Luyan 34 | irrigation cultivation soil | 8.25 | 44°0'44.00" N | 87°21'40.20" E | 375 | flowering stage | Wusu |
| WTBT | 3 | Luyan 34 | irrigation cultivation soil | 8.21 | 44°0'44.00" N | 87°21'40.20" E | 375 | boll-opening stage | Wusu |
| JMBT | 3 | Hexin 26 | desert grey soil | 8.15 | 44°34'57.07"N | 82°24'37.26" E | 326 | seedling stage | Jinghe |
| JLBT | 3 | Hexin 26 | desert grey soil | 8.24 | 44°34'57.07"N | 82°24'37.26" E | 326 | bud stage | Jinghe |
| JHBT | 3 | Hexin 26 | desert grey soil | 8.3 | 44°34'57.07"N | 82°24'37.26" E | 326 | flowering stage | Jinghe |
| JTBT | 3 | Hexin 26 | desert grey soil | 8.08 | 44°34'57.07"N | 82°24'37.26" E | 326 | boll-opening stage | Jinghe |
