# Supplementary material for: Differentiation and Variability in the Rhizosphere and Endosphere Microbiomes of Healthy and Diseased Cotton (Gossypium sp.)
Source: Front Microbiol. 2021 Dec 6;12:765269. doi: 10.3389/fmicb.2021.765269 (PMC8685383; doi:10.3389/fmicb.2021.765269)
Supplement: Supplementary file 2 [file Table_2.docx]

**Table S2** Cotton Variety, locations, elevations, pH and soil types

| Samples | Number of sample | Variety | Soil  classification | Soil pH | Latitude | longitude | Elevation (m) | The growth period | Locales |
| --- | --- | --- | --- | --- | --- | --- | --- | --- | --- |
| AMJZ | 3 | Zhong 35 | irrigated desert soil | 8.20 | 40°20'19.90"N | 81°11'2.76" E | 998 | seedling stage | Alaer |
| ALJZ | 3 | Zhong 35 | irrigated desert soil | 8.17 | 40°20'19.90"N | 81°11'2.76" E | 998 | bud stage | Alaer |
| AHJZ | 3 | Zhong 35 | irrigated desert soil | 8.12 | 40°20'19.90"N | 81°11'2.76" E | 998 | flowering stage | Alaer |
| ATJZ | 3 | Zhong 35 | irrigated desert soil | 8.08 | 40°20'19.90"N | 81°11'2.76" E | 998 | boll-opening stage | Alaer |
| KMJZ | 3 | Zhong 49 | desert grey soil | 7.97 | 40°27'55.80" N | 87°7'53.40"E | 851 | seedling stage | Kuerle |
| KLJZ | 3 | Zhong 49 | desert grey soil | 8.14 | 40°27'55.80" N | 87°7'53.40"E | 851 | bud stage | Kuerle |
| KHJZ | 3 | Zhong 49 | desert grey soil | 8.18 | 40°27'55.80" N | 87°7'53.40"E | 851 | flowering stage | Kuerle |
| KTJZ | 3 | Zhong 49 | desert grey soil | 8.12 | 40°27'55.80" N | 87°7'53.40"E | 851 | boll-opening stage | Kuerle |
| TMJZ | 3 | Xinluzhong 60 | alluvial soil | 8.03 | 39°55'12.50" N | 79°7'41.81" E | 1091 | seedling stage | Tumushuke |
| TLJZ | 3 | Xinluzhong 60 | alluvial soil | 8.13 | 39°55'12.50" N | 79°7'41.81" E | 1091 | bud stage | Tumushuke |
| THJZ | 3 | Xinluzhong 60 | alluvial soil | 8.13 | 39°55'12.50" N | 79°7'41.81" E | 1091 | flowering stage | Tumushuke |
| TTJZ | 3 | Xinluzhong 60 | alluvial soil | 8.07 | 39°55'12.50" N | 79°7'41.81" E | 1091 | boll-opening stage | Tumushuke |
| HMJZ | 3 | Mihe 1 | brown desert soil | 7.87 | 42°54'52.09" N | 93°16'14.99" E | 875 | seedling stage | Hami |
| HLJZ | 3 | Mihe 1 | brown desert soil | 8.08 | 42°54'52.09" N | 93°16'14.99" E | 875 | bud stage | Hami |
| HHJZ | 3 | Mihe 1 | brown desert soil | 8.25 | 42°54'52.09" N | 93°16'14.99" E | 875 | flowering stage | Hami |
| HTJZ | 3 | Mihe 1 | brown desert soil | 8.4 | 42°54'52.09" N | 93°16'14.99" E | 875 | boll-opening stage | Hami |
| SMJZ | 3 | 15-19 | desert grey soil | 8.20 | 44°20'6.29"N | 86°2'51.76" E | 421 | seedling stage | Shihezi |
| SLJZ | 3 | 15-19 | desert grey soil | 8.14 | 44°20'6.29"N | 86°2'51.76" E | 421 | bud stage | Shihezi |
| SHJZ | 3 | 15-19 | desert grey soil | 8.17 | 44°20'6.29"N | 86°2'51.76" E | 421 | flowering stage | Shihezi |
| STJZ | 3 | 15-19 | desert grey soil | 7.92 | 44°20'6.29"N | 86°2'51.76" E | 421 | boll-opening stage | Shihezi |
| WMJZ | 3 | Luyan 34 | irrigation cultivation soil | 8.03 | 44°0'44.00" N | 87°21'40.20" E | 375 | seedling stage | Wusu |
| WLJZ | 3 | Luyan 34 | irrigation cultivation soil | 8.03 | 44°0'44.00" N | 87°21'40.20" E | 375 | bud stage | Wusu |
| WHJZ | 3 | Luyan 34 | irrigation cultivation soil | 8.25 | 44°0'44.00" N | 87°21'40.20" E | 375 | flowering stage | Wusu |
| WTJZ | 3 | Luyan 34 | irrigation cultivation soil | 8.21 | 44°0'44.00" N | 87°21'40.20" E | 375 | boll-opening stage | Wusu |
| JMJZ | 3 | Hexin 26 | desert grey soil | 8.15 | 44°34'57.07"N | 82°24'37.26" E | 326 | seedling stage | Jinghe |
| JLJZ | 3 | Hexin 26 | desert grey soil | 8.24 | 44°34'57.07"N | 82°24'37.26" E | 326 | bud stage | Jinghe |
| JHJZ | 3 | Hexin 26 | desert grey soil | 8.3 | 44°34'57.07"N | 82°24'37.26" E | 326 | flowering stage | Jinghe |
| JTJZ | 3 | Hexin 26 | desert grey soil | 8.08 | 44°34'57.07"N | 82°24'37.26" E | 326 | boll-opening stage | Jinghe |
| AMBZ | 3 | Zhong 35 | irrigated desert soil | 8.20 | 40°20'19.90"N | 81°11'2.76" E | 998 | seedling stage | Alaer |
| ALBZ | 3 | Zhong 35 | irrigated desert soil | 8.17 | 40°20'19.90"N | 81°11'2.76" E | 998 | bud stage | Alaer |
| AHBZ | 3 | Zhong 35 | irrigated desert soil | 8.12 | 40°20'19.90"N | 81°11'2.76" E | 998 | flowering stage | Alaer |
| ATBZ | 3 | Zhong 35 | irrigated desert soil | 8.08 | 40°20'19.90"N | 81°11'2.76" E | 998 | boll-opening stage | Alaer |
| KMBZ | 3 | Zhong 49 | desert grey soil | 7.97 | 40°27'55.80" N | 87°7'53.40"E | 851 | seedling stage | Kuerle |
| KLBZ | 3 | Zhong 49 | desert grey soil | 8.14 | 40°27'55.80" N | 87°7'53.40"E | 851 | bud stage | Kuerle |
| KHBZ | 3 | Zhong 49 | desert grey soil | 8.18 | 40°27'55.80" N | 87°7'53.40"E | 851 | flowering stage | Kuerle |
| KTBZ | 3 | Zhong 49 | desert grey soil | 8.12 | 40°27'55.80" N | 87°7'53.40"E | 851 | boll-opening stage | Kuerle |
| TMBZ | 3 | Xinluzhong 60 | alluvial soil | 8.03 | 39°55'12.50" N | 79°7'41.81" E | 1091 | seedling stage | Tumushuke |
| TLBZ | 3 | Xinluzhong 60 | alluvial soil | 8.13 | 39°55'12.50" N | 79°7'41.81" E | 1091 | bud stage | Tumushuke |
| THBZ | 3 | Xinluzhong 60 | alluvial soil | 8.13 | 39°55'12.50" N | 79°7'41.81" E | 1091 | flowering stage | Tumushuke |
| TTBZ | 3 | Xinluzhong 60 | alluvial soil | 8.07 | 39°55'12.50" N | 79°7'41.81" E | 1091 | boll-opening stage | Tumushuke |
| HMBZ | 3 | Mihe 1 | brown desert soil | 7.87 | 42°54'52.09" N | 93°16'14.99" E | 875 | seedling stage | Hami |
| HLBZ | 3 | Mihe 1 | brown desert soil | 8.08 | 42°54'52.09" N | 93°16'14.99" E | 875 | bud stage | Hami |
| HHBZ | 3 | Mihe 1 | brown desert soil | 8.25 | 42°54'52.09" N | 93°16'14.99" E | 875 | flowering stage | Hami |
| HTBZ | 3 | Mihe 1 | brown desert soil | 8.4 | 42°54'52.09" N | 93°16'14.99" E | 875 | boll-opening stage | Hami |
| SMBZ | 3 | 15-19 | desert grey soil | 8.20 | 44°20'6.29"N | 86°2'51.76" E | 421 | seedling stage | Shihezi |
| SLBZ | 3 | 15-19 | desert grey soil | 8.14 | 44°20'6.29"N | 86°2'51.76" E | 421 | bud stage | Shihezi |
| SHBZ | 3 | 15-19 | desert grey soil | 8.17 | 44°20'6.29"N | 86°2'51.76" E | 421 | flowering stage | Shihezi |
| STBZ | 3 | 15-19 | desert grey soil | 7.92 | 44°20'6.29"N | 86°2'51.76" E | 421 | boll-opening stage | Shihezi |
| WMBZ | 3 | Luyan 34 | irrigation cultivation soil | 8.03 | 44°0'44.00" N | 87°21'40.20" E | 375 | seedling stage | Wusu |
| WLBZ | 3 | Luyan 34 | irrigation cultivation soil | 8.03 | 44°0'44.00" N | 87°21'40.20" E | 375 | bud stage | Wusu |
| WHBZ | 3 | Luyan 34 | irrigation cultivation soil | 8.25 | 44°0'44.00" N | 87°21'40.20" E | 375 | flowering stage | Wusu |
| WTBZ | 3 | Luyan 34 | irrigation cultivation soil | 8.21 | 44°0'44.00" N | 87°21'40.20" E | 375 | boll-opening stage | Wusu |
| JMBZ | 3 | Hexin 26 | desert grey soil | 8.15 | 44°34'57.07"N | 82°24'37.26" E | 326 | seedling stage | Jinghe |
| JLBZ | 3 | Hexin 26 | desert grey soil | 8.24 | 44°34'57.07"N | 82°24'37.26" E | 326 | bud stage | Jinghe |
| JHBZ | 3 | Hexin 26 | desert grey soil | 8.3 | 44°34'57.07"N | 82°24'37.26" E | 326 | flowering stage | Jinghe |
| JTBZ | 3 | Hexin 26 | desert grey soil | 8.08 | 44°34'57.07"N | 82°24'37.26" E | 326 | boll-opening stage | Jinghe |
