# Supplementary figures and images for: Differentiation and Variability in the Rhizosphere and Endosphere Microbiomes of Healthy and Diseased Cotton (Gossypium sp.)
Source: Front Microbiol. 2021 Dec 6;12:765269. doi: 10.3389/fmicb.2021.765269 (PMC8685383; doi:10.3389/fmicb.2021.765269)

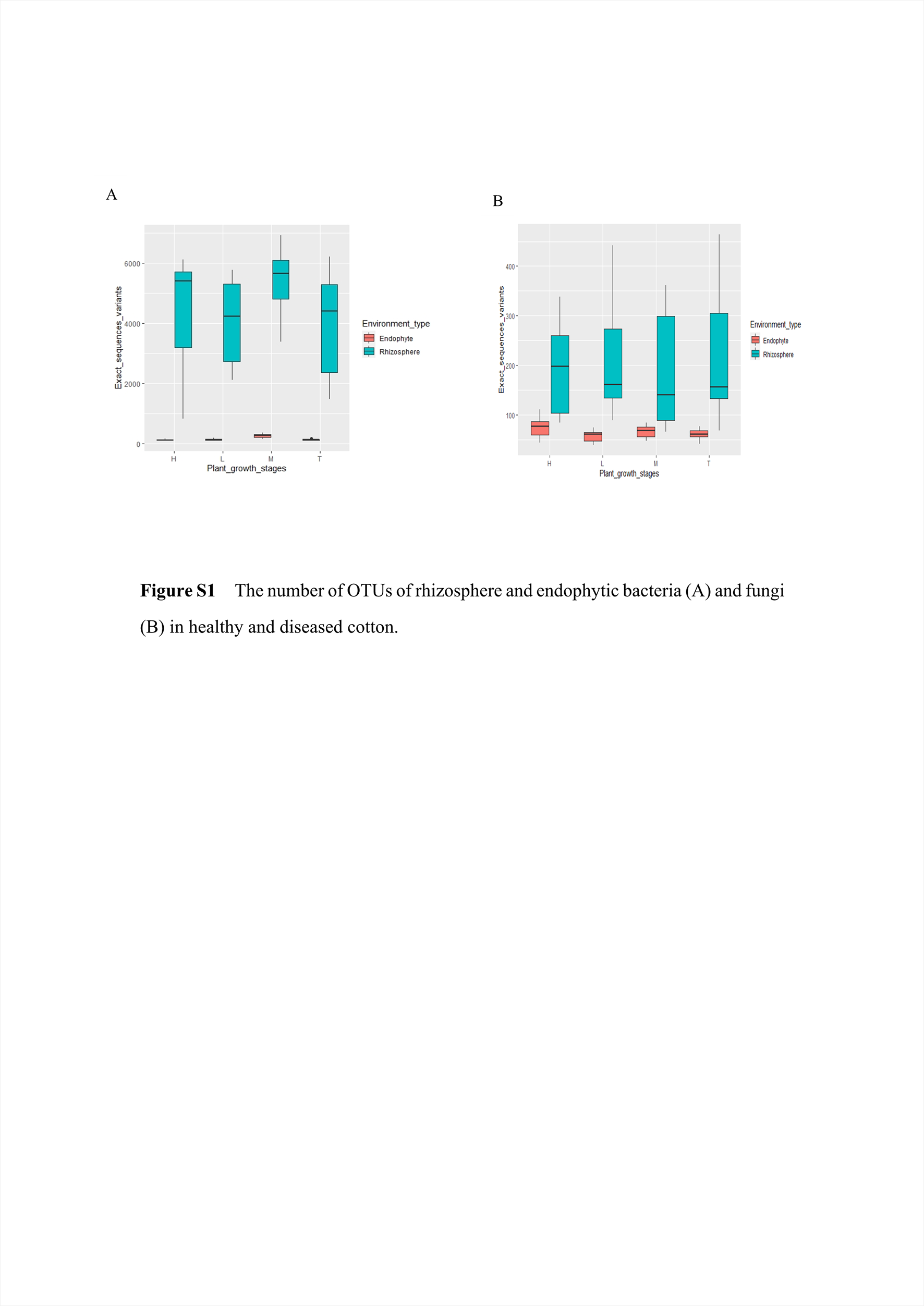

Supplement: Supplementary file 3 [file Image_1.tif]

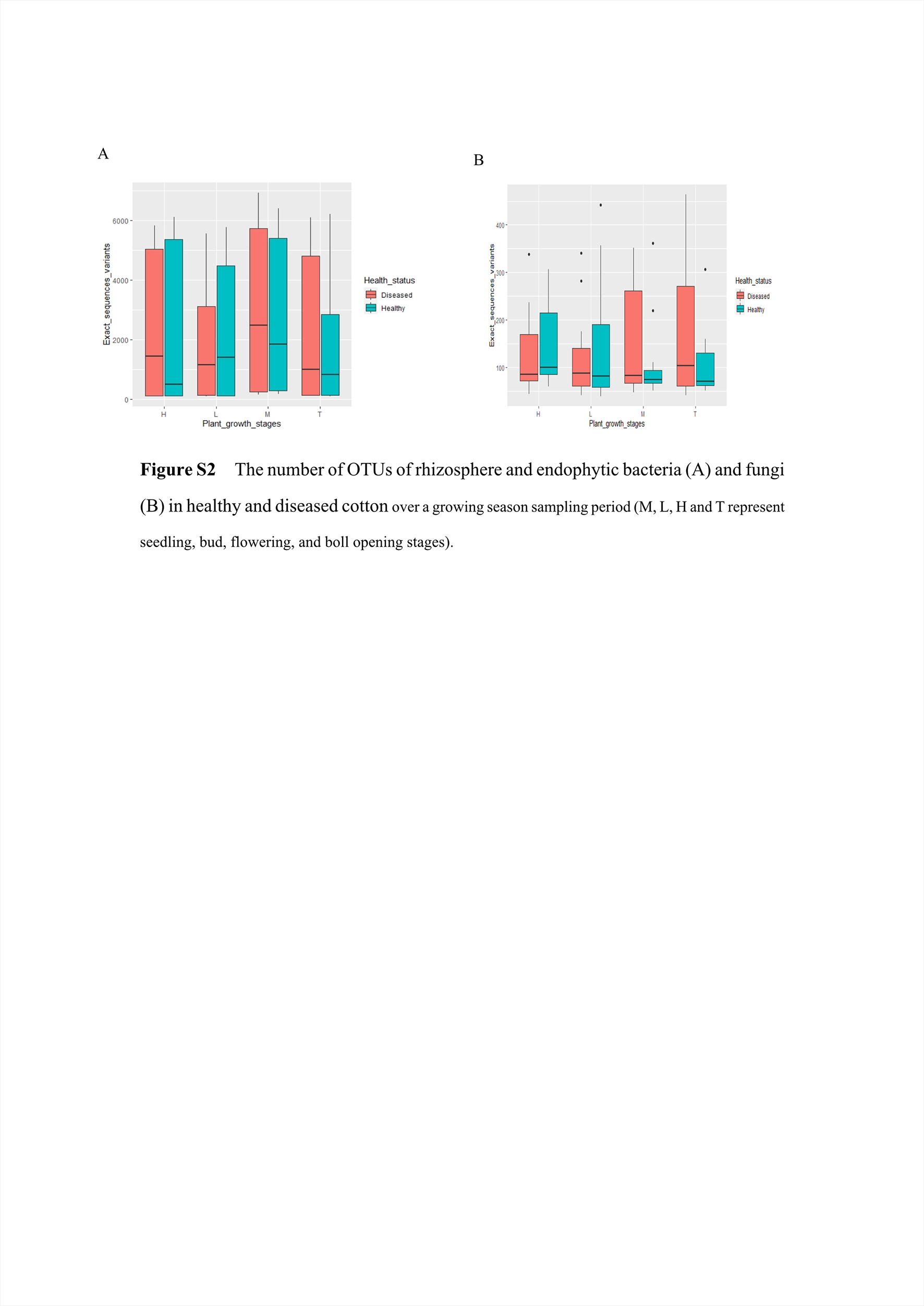

Supplement: Supplementary file 4 [file Image_2.tif]

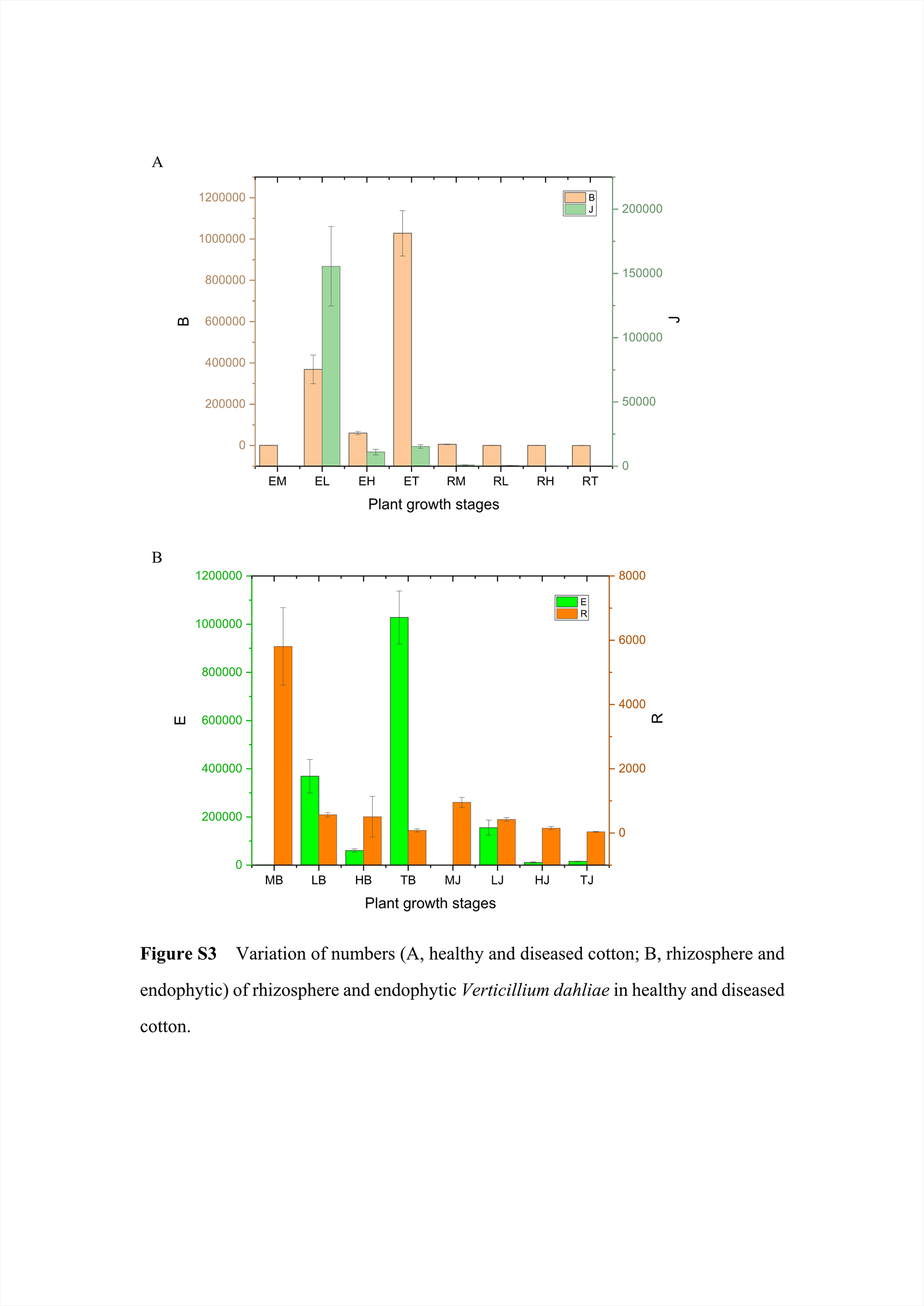

Supplement: Supplementary file 5 [file Image_3.tif]

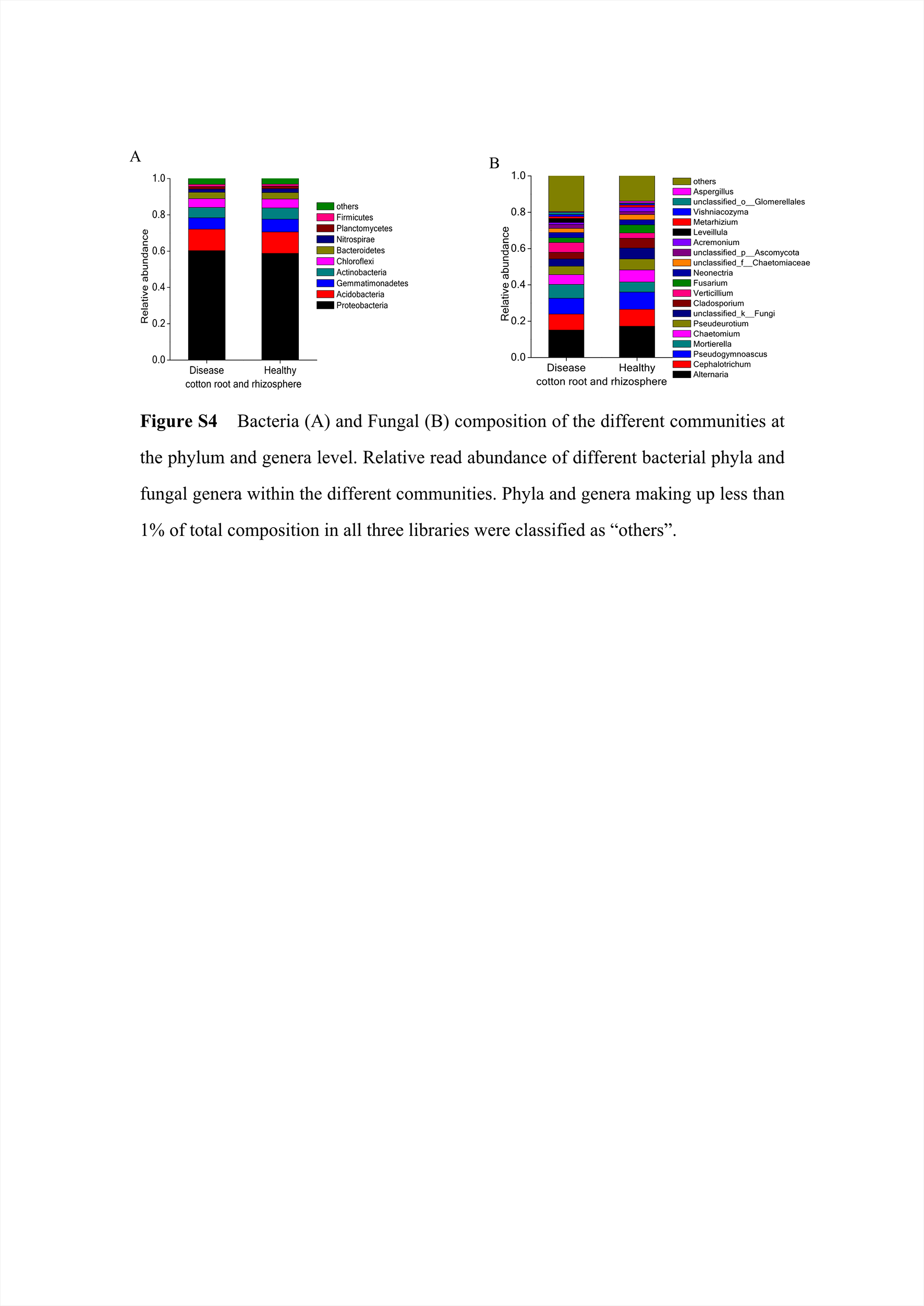

Supplement: Supplementary file 6 [file Image_4.tif]

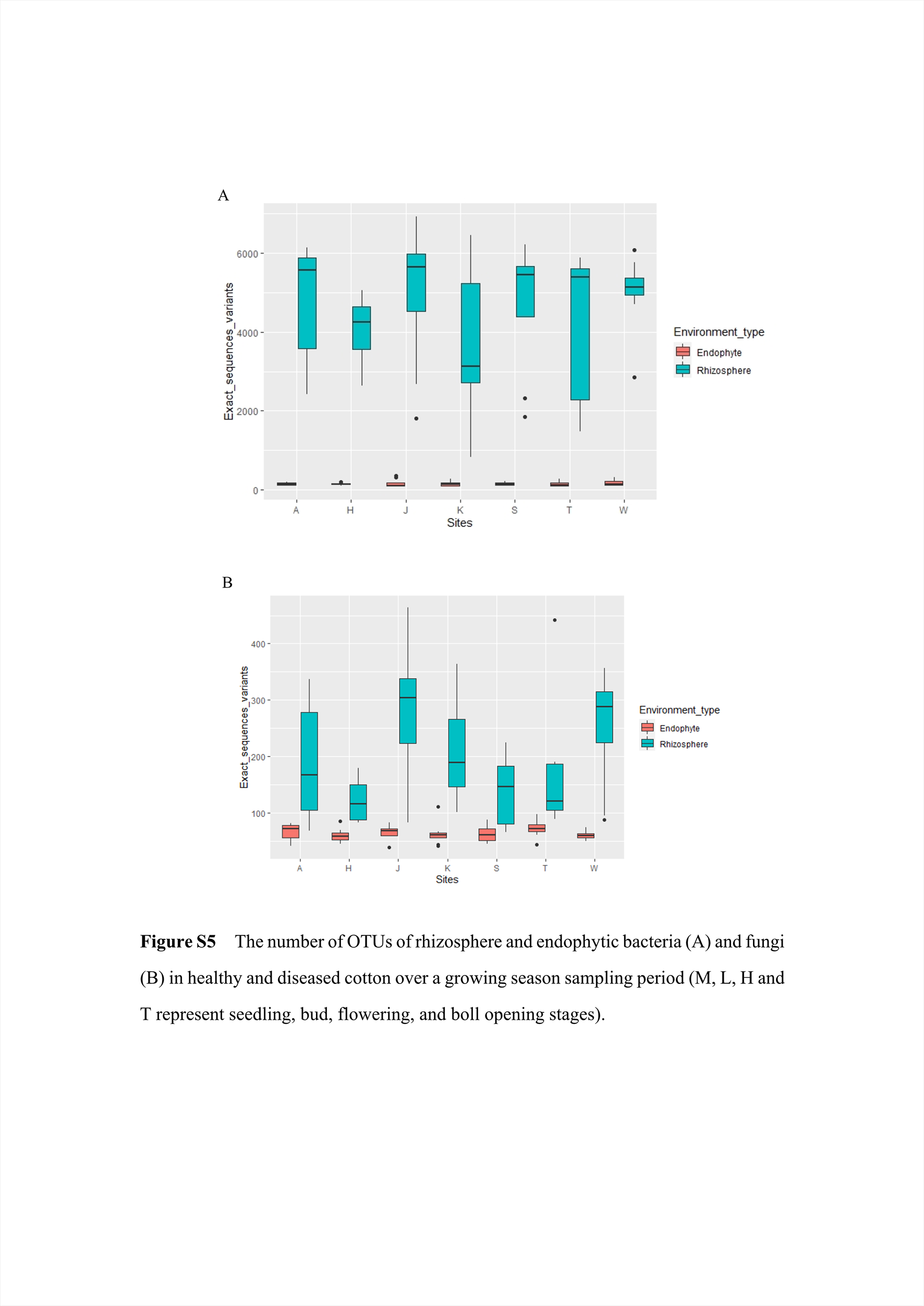

Supplement: Supplementary file 7 [file Image_5.TIF]

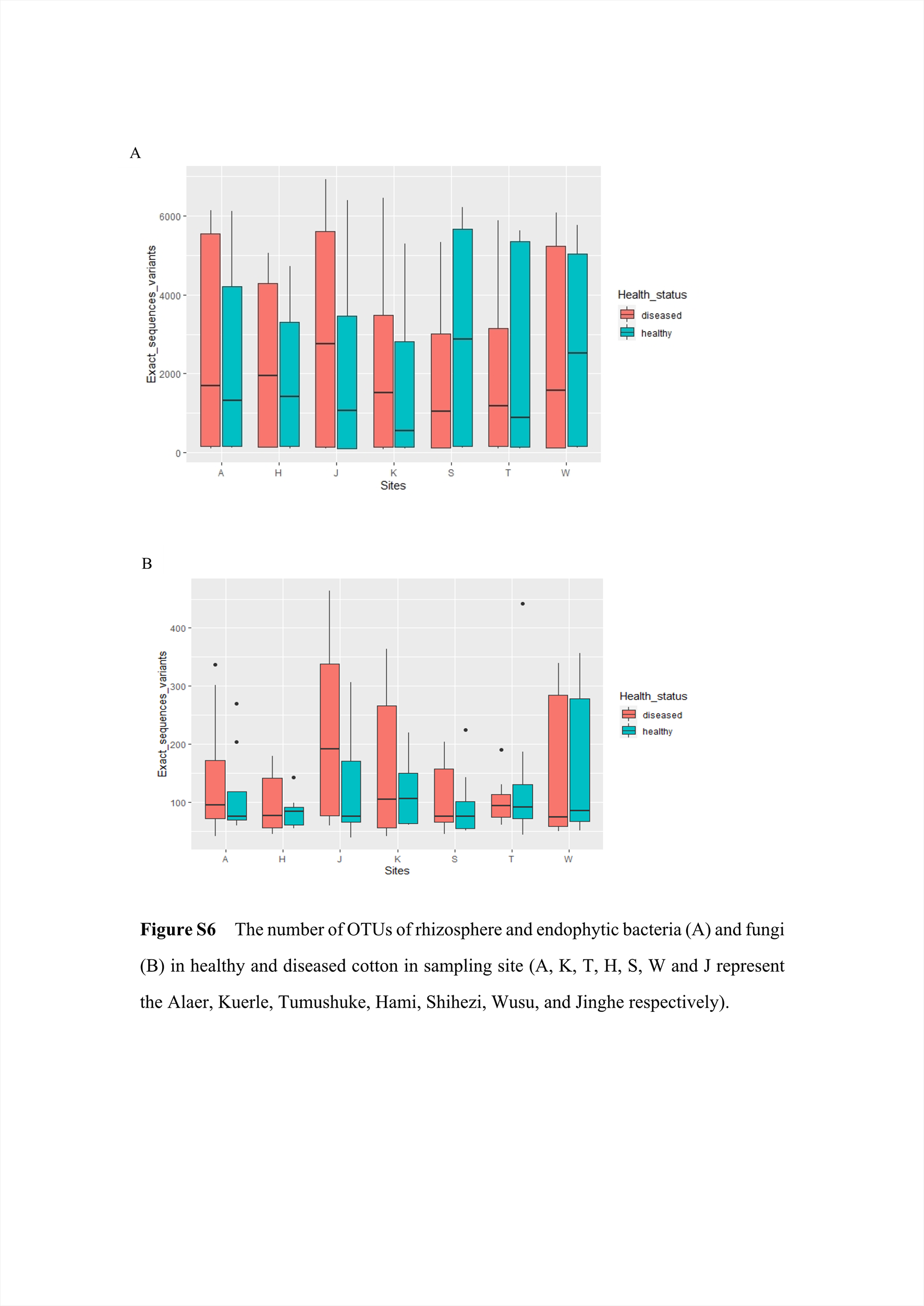

Supplement: Supplementary file 8 [file Image_6.tif]
